# Supplementary material for: Evaluating genetic ancestry and self-reported ethnicity in the context of carrier screening
Source: BMC Genet. 2017 Nov 28;18:99. doi: 10.1186/s12863-017-0570-y (PMC5704547; doi:10.1186/s12863-017-0570-y)
Supplement: Supplementary file 1 — Requisition form used in this study. (PDF 287 kb) [file 12863_2017_570_MOESM1_ESM.pdf]

Recombine

LEAVING ANY SECTION BLANK OR INCOMPLETE WILL RESULT IN A DELAY OR CANCELLATION OF TESTING

CarrierMap
Test Requisition Form

Patient, please complete this column:

PATIENT INFORMATION

First Name ____________________ Last Name ____________________

Gender: ☐ Male ☐ Female DOB (mm/dd/yy) ____________

Ethnicity: (Check all that apply) ☐ African ☐ East Asian ☐ European ☐ French Canadian ☐ Jewish ☐ Latin American ☐ Mediterranean ☐ Middle Eastern ☐ Native American ☐ South Asian ☐ Southeast Asian ☐ Other________

Address ______________________________

City ____________________ State ______ Zip ________

Phone ______________________________

Email ______________________________
Required to receive results and schedule genetic counseling

Partner Information

By providing your partner's name and date of birth, you are giving Recombine permission to link your results with your partner's results.

Partner's Name ______________________________

Partner's DOB (mm/dd/yy) ______________________________

BILLING INFORMATION

☐ Option A: Bill My Insurance (Insurance Information & Credit Card Information Required)

☐ Option B: Self Pay/No Insurance (Credit Card Information Required)

Insurance Information

In addition to completing the fields below, please attach a copy of the front and back of your insurance card or email a picture to insurance@recombine.com.

Insurance Company Name ______________________________

Policyholder Name ______________________________

Policy Number / Member ID ______________________________

Policyholder DOB (mm/dd/yy) ______________________________

Relation to Patient: ☐ Self ☐ Spouse ☐ Dependent ☐ Other

Credit Card Information

Card # ______________________________

Security Code ____________ Expiration Date (mm/yy) ____________

PATIENT CONSENT

Research Consent

Upon completion of my genetic testing, and with my authorization, Recombine may store the remaining sample and test data to perform research studies. Recombine may keep my sample and test data for as long as Recombine deems it useful for research purposes. Recombine will remove all personal information from my test data and sample prior to using my information for such studies. My name or other personal identifying information will not be used or linked to the results of any research studies and publications. This authorization will not expire, but I may revoke my authorization at any time by contacting Recombine at 855-687-4363. Any revocation of this authorization will not affect research studies performed on my sample and test data prior to my revocation. Recombine may not condition genetic testing on my decision to authorize or not authorize the use of my remaining sample and test information for research.

☐ I authorize Recombine to store my patient sample and test data to perform research studies as described above and on the attached informed consent form.

☐ I do not authorize Recombine to store and use my patient sample and test data for research studies.

Test Authorization

By signing below, I certify that I have read the attached informed consent form for genetic testing, have discussed the benefits, risks, and limitations of genetic testing with my physician, and consent for Recombine to perform the genetic test(s) ordered by my physician. I further certify that I have received and have reviewed a copy of "Patient Billing Basics", and have been given the opportunity to ask my physician and Recombine questions concerning the payment terms outlined within this document. I agree that all of my questions have been answered to my satisfaction and I accept the payment terms.

Patient Signature ______________________________ Date ____________

Clinic staff, please complete this column:

CLINIC INFORMATION

Ordering Physician

SPECIMEN INFORMATION

Collection Date (mm/dd/yy) ____________ Patient ID # ____________

Specimen Type: ☐ Saliva ☐ Blood ☐ Semen ☐ Extracted DNA

SELECT ANY THAT APPLY

☐ Patient is a donor

☐ Bill clinic for testing

☐ Patient is pregnant (specify gestational age) ______________________

☐ Patient is a known carrier (specify disease) ______________________

☐ Sample redraw

TEST INFORMATION

CarrierMap Genotyping Test Panel (select one)

Additional Testing (additional charges apply)

☐ Gene Sequencing
To order sequencing, please check the box above, and select the diseases/genes to be sequenced on the enclosed Sequencing Order Form.

TEST INDICATION

ICD-10 Codes – CHECK ALL THAT APPLY

☐ Z15.89	High Risk Ethnicity
☐ Z31.430	Screening for genetic disease carrier status - Female
☐ Z31.440	Screening for genetic disease carrier status - Male
☐ Z34.01	Supervision, normal first pregnancy 1st Trimester
☐ Z34.02	Supervision, normal first pregnancy 2nd Trimester
☐ Z34.03	Supervision, normal first pregnancy 3rd Trimester
☐ Z34.81	Supervision, other normal pregnancy 1st Trimester
☐ Z34.82	Supervision, other normal pregnancy 2nd Trimester
☐ Z34.83	Supervision, other normal pregnancy 3rd Trimester
☐ Z84.3	Consanguinity
☐ Z84.81	Family History of Genetic Disease Carrier ____________ (specify disease & family member)
☐ OTHERS:	____________

ORDERING HEALTH CARE PROFESSIONAL AUTHORIZATION

I hereby order Recombine to conduct the above tests, which I have determined to be medically necessary. I have explained genetic testing (including the risks, benefits, and alternatives) to this individual. I have addressed the limitations outlined in the informed consent form, and I have answered this person's questions to the best of my ability.

Provider Signature ______________________________ Date ____________

Instructions for Sample Collection

1. Complete enclosed requisition and consent forms in their entirety. (If testing the patient's partner, be sure to complete the "Partner Information" section).
2. Fill out patient's name and DOB on the patient identifier stickers below. Place one patient identifier sticker on the sample collection tube.
3. Upon collecting the patient's sample, place the collection tube in the biohazard bag.
4. Place the kit in the provided FedEx Clinical Pak. Ship by dropping the kit at a FedEx drop box or scheduling a pick-up through FedEx.com or 1-800-goFedEx.

For sample collection tube	Extra sticker, if needed	Extra sticker, if needed	Extra sticker, if needed
Patient First & Last Name: ____________	Patient First & Last Name: ____________	Patient First & Last Name: ____________	Patient First & Last Name: ____________
Date of Birth: ____________ (mm/dd/yy)	Date of Birth: ____________ (mm/dd/yy)	Date of Birth: ____________ (mm/dd/yy)	Date of Birth: ____________ (mm/dd/yy)

Copyright 2015 Recombine, Inc.
