# Supplementary figures and images for: Evaluating genetic ancestry and self-reported ethnicity in the context of carrier screening
Source: BMC Genet. 2017 Nov 28;18:99. doi: 10.1186/s12863-017-0570-y (PMC5704547; doi:10.1186/s12863-017-0570-y)

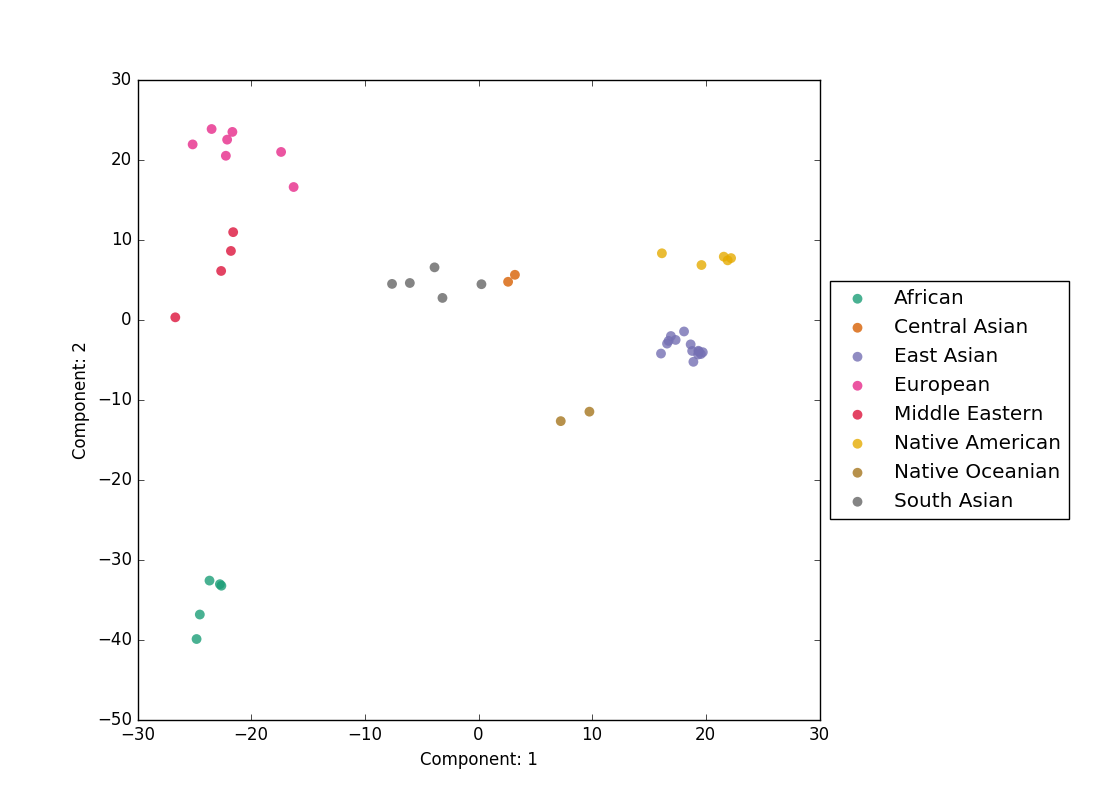

Supplement: Supplementary file 3 — Plot of the first and second principal components obtained by Principal Component Analysis on 44 geographic groups (described in Additional file 2: Table S1) and 1142 AIMs. Each geographic group is shown as a point and is colored according to the continental group to which it belongs. The plot illustrates that the AIMs separate most continental groups well, but the Middle Eastern and Central Asian groups do not form distinct clusters. (PNG 49 kb) [file 12863_2017_570_MOESM3_ESM.png]
